# An Association of Framingham Risk Score with Patient Determined Disease Steps in a Cohort of Relapsing-Remitting Multiple Sclerosis Patients: An Italian Real-World Monocentric Experience

**DOI:** 10.2174/1570159X22666240815120018

**Published:** 2024-08-15

**Authors:** Aurora Zanghì, Paola Sofia Di Filippo, Carlo Avolio, Emanuele D’Amico

**Affiliations:** 1 Department of Medical and Surgical Sciences, University of Foggia, Foggia, Italy

**Keywords:** Framingham risk score, multiple sclerosis, patient determined disease steps, disability, monocentric experience, cardiovascular diseases

## Abstract

**Background:**

The associations between Multiple Sclerosis (MS) and cardiovascular diseases, drawn from epidemiological studies, have attracted much attention in recent years.

**Materials and Methods:**

The present study employed a monocentric, observational, retrospective cohort design. The primary objective of the study was to describe the Framingham Risk Score (FRS) rate in a cross-sectional analysis of our cohort of relapsing-remitting MS patients who are regularly followed up and, if applicable, to identify any association with the patient's Patient Determined Disease Steps (PDDS). Cardiovascular risk was classified as follows: low if the FRS is less than 10%, moderate if it is 10% to 19%, and high if it is 20% or higher.

**Results:**

A total cohort of 229 patients was enrolled. The sample consists of 163 women (71.2%). FRS categories were distributed as follows: 97 (42.3%) patients had low FRS, 84 (36.7%) patients had moderate FRS, and 48 (21%) patients had high FRS. In the univariable ordinal regression analysis, one one-point increase in the PDDS scale was associated with a 24% risk of high FRS (*vs*. low) (proportional odds ratio [OR]=2.426, 95% confidence interval [CI] 1.660-3.545; *p* <.0001). The results were also confirmed by the EDSS score, with a point increase in the EDSS score associated with a 19% risk of high FRS (*vs*. low) (proportional OR=1.953, 95% CI 1.429-2.669-1.04; *p* <.0001).

**Conclusion:**

The FRS demonstrated an association with the patient's “perception of the disease” as indicated by the PDDS. Further studies with larger cohorts are needed to adequately address cardiovascular risk in life-threatening conditions, such as MS.

## 1 INTRODUCTION

Multiple sclerosis (MS) is a chronic disease characterized by inflammatory and degenerative processes within the central nervous system (CNS) [1, 2]. The progression of MS leads to persistent disability and a diminished quality of life, often exacerbated by concurrent comorbidities [3, 4].

The associations between MS and cardiovascular diseases, as observed in epidemiological studies, have garnered significant attention in recent years [5-7]. MS patients exhibit a higher burden of cardiovascular comorbidities compared to the general population [8].

Notably, hypertension, ischemic heart disease, excessive body weight, and disorders of cerebral and peripheral arteries have been correlated with increased disability, relapse rates, and disease course of MS [9, 10]. Given their substantial prevalence in the MS population, cardiovascular comorbidities have become a focal point of investigation.

Traditional observational studies are susceptible to potential confounders and reverse causality bias, making it difficult to infer causality [11, 12]. Therefore, the potential causal relationship between MS and cardiovascular risks remains unclear.

The Framingham risk score (FRS) is a cardiovascular risk scoring system that estimates the probability of developing cardiovascular disease within 10 years [13]. Previous studies have utilized FRS in the context of MS and have associated it with MS-related disability and disease trajectory (*i.e*., relapsing or progressive) [10, 14, 15].

However, data on patient-reported outcomes (PROs) and cardiovascular comorbidities have not been fully investigated. The Patient Determined Disease Steps (PDDS) is a promising PRO measure of disability that is simple and reliable [16-18]. While FRS has been studied in MS populations, no studies have focused on quality of life or patients' perceptions of their disease. Coexisting health issues directly impact the quality of life of MS patients, and adopting an overly aggressive treatment approach may impede the progression of MS itself [19, 20]. The lack of extensive and uniformly standardized epidemiological investigations addressing the issues outlined in this report represents a major obstacle to the optimal management of individuals with MS.

In this cross-sectional monocentric study, our objective was to profile FRS within our Relapsing-Remitting MS (RRMS) cohort and explore any potential correlations with PDDS and disease characteristics.

## 2 MATERIALS AND METHODS

### 2.1 Database and Study Population

The present study employed a monocentric, observational, retrospective cohort design. All patients admitted to the MS Centre in Foggia, Italy, from January 1^st^ to June 30^th^, 2023, were sequentially invited to participate in the study.

Key eligibility criteria were: (1) age > 18 years; 2) diagnosis of RRMS according to the 2017 McDonald criteria [21].

The minimum dataset for all enrolled patients included demographic data (sex, date of birth) and clinical data, which consisted of the year of diagnosis, naive/switcher status, relapses, the Expanded Disability Status Scale (EDSS) assessed by a certified neurologist [22].

The patients undergoing their first disease-modifying treatment (DMT), and thus not exposed to others, have been categorized as “naïve”. Conversely, patients exposed to more than one DMT throughout their clinical history have been categorized as “switchers”.

During the visit, all the patients were allowed to ultimate the PDDS, which is a valid PRO of disability in MS [17, 23, 24].

MRI information was collected in terms of the total count of T1-gadolinium-enhancing brain lesions and or a new or newly enlarging T2 brain lesion.

Data entry was performed using iMed^©^ software, and data were extracted in June 2023.

### 2.2 Procedures and Outcomes

#### 2.2.1 Cardiovascular Risk Evaluation

The primary variable of interest in this investigation was the FRS, a predictive tool for assessing vascular disease risk that considers the varying significance of contributing factors. The FRS is a gender-specific composite index that integrates data on age, smoking history, hypertension, and diabetes, in addition to either lipid profile or body mass index (BMI) [25]. Positive scores are assigned to risk factors associated with increased cardiovascular risk, such as a history of smoking, while negative scores are allocated to protective factors, including elevated high-density lipoprotein levels. Specifically, we documented smoking habits, classifying individuals who engaged in regular smoking over the past 12 months as smokers.

Height and weight were assessed using standardized hospital clinical procedures, and BMI was computed. Blood pressure was measured twice after the participant had been seated for at least 5 minutes, and the average was utilized for analysis. The presence of Type II diabetes was determined if the participant was undergoing treatment with insulin or oral hypoglycemic agents or if fasting blood glucose levels exceeded 126 mg/dl in previous blood examinations (all subjects underwent at least two different blood tests within the previous 12 months, in accordance with clinical practice). Due to the unavailability of serum lipid measurements for all patients, we applied the version of the FRS that relies on BMI, utilizing measurements obtained at the latest follow-up visit [25].

### 2.3 Study Endpoints

The primary study outcome was to describe in a cross-sectional fashion the FRS rate in our cohort of RRMS regularly followed up and then, if any, to find an association with PDDS and then with disease characteristics. Cardiovascular risk was classified as follows: low if the FRS is less than 10%, moderate if it is 10% to 19%, and high if it is 20% or higher.

We categorized as active patients those who had clinical disease activity (relapses) or MRI activity in the year before the index visit.

A relapse was defined as the development of new symptoms or exacerbation of existing symptoms that persisted for ≥ 24 hours, in the absence of concurrent illness or fever, and occurred ≥ 30 days after a previous relapse.

MRI activity was considered as a new T1-gadolinium-enhancing brain lesion and or a new or newly enlarging T2 brain lesion.

The overweight range is classified as a BMI of 25.0 to < 30, while a BMI of 30.0 or higher falls within the obesity range.

### 2.4 Statistical Analysis

All the patients ‘characteristics summary statistics are reported in terms of frequencies (%) for categorical variables, mean standard deviation (S.D.), or median with interquartile range (IQR) for continuous variables. The Kolmogorov test was used to verify data distribution. According to this latter, a parametric or nonparametric test was employed. The Bonferroni test was used for post hoc analysis.

According to the Akaike information criterion, we selected the model with the best statistical inferential properties. All the models were estimated using Breslow’s tie correction. The data are described according to the nature of the variables. The chi-squared test was applied when necessary to evaluate the association between categorical variables. Student's t-test or a non-parametric Mann-Whitney U test was applied according to the data distribution.

The association between FRS and disease characteristics was investigated using an ordinal logistic regression model (low risk, intermediate risk, and high risk) to obtain proportional odds. The assumption of ordinal logistic regression was verified using parallel lines test.

The results are presented as odds ratios (ORs) and the corresponding 95% confidence intervals (95% CIs).

The covariates inserted were sex, disease duration, naïve/switcher status regarding DMTs (as dichotomous), disease activity in the year before the index date (as dichotomous), EDSS (index date), and PDSS (index date). After verification of multicollinearity, age, and sex were not included in the multivariable model.

Multicollinearity was evaluated using the variance inflation factor, where a value > 10 was considered an index of collinearity amongst variables. Due to the multicollinearity between “FRS” variables and age (variance inflation factor > 10), age was not added to the univariable model.

Additionally, PDDS and EDSS also had a variance inflation factor > 10, so these two variables were not added to the multivariable model.

Variables with a *p*-value < 0.10 in the univariable analysis were considered to build the multivariable model.

SPSS version 21.0 was used for all analyses (IBM SPSS Statistics 21, IBM^©^, Armonk, NY, USA).

### 2.5 Protocol Approval Standards, Registrations, and Patient Consent

The study protocol was approved by the local ethics committee (Ethics Committee of Foggia, Italy) (N.14/CE/2022).

Patients provided written informed consent. The study was conducted in accordance with the ethical principles of the Declaration of Helsinki and with the appropriate national regulations.

## 3 RESULTS

**Fig. (1):**
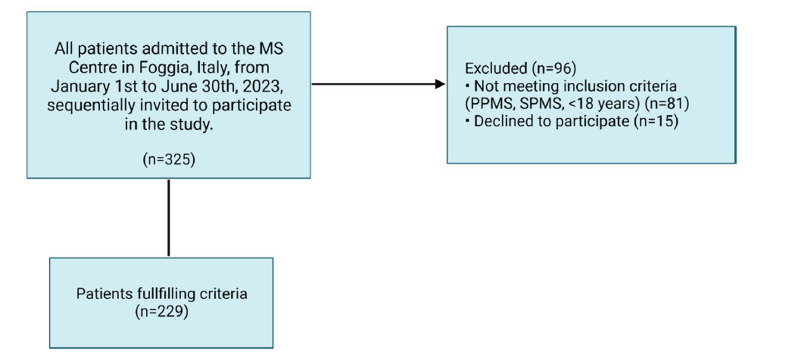
Flow chart of the study. **Abbreviations:** RRMS, relapsing-remitting Multiple Sclerosis; PPMS, progressive Multiple Sclerosis; SPMS, secondary progressive Multiple Sclerosis.

From a total cohort of 358 patients who had access to the MS centre during the index window, 229 were enrolled. Out of them, 163 (71.2%) were women, with a mean age of 40.4 ± 12.6 years (Fig. **1**). The baseline characteristics of the entire sample are reported in Table **1**.

Disease activity in the year before enrolment was recorded in 45 patients (19.6%).

FRS categories were distributed as follows: 97 (42.3%) patients had low FRS, 84 (36.7%) patients had moderate FRS, and 48 (21%) patients had high FRS.

Patients with high FRS had higher EDSS (index date) and PDDS than the other two categories (both *p <*.000) (Table **2**).

In the univariable ordinal regression analysis, a PDDS one-point increase was associated with a 24% risk of high FRS (*vs*. low) (proportional OR=2.426, 95% CI 1.660-3.545; *p <*.0001) (Table **3**). The PDSS score showed no association with moderate FRS (*vs*. low) (proportional OR =.677, 95% CI .312-1.471, *p* =.324).

When other variables were analysed, a point increase in the EDSS score recorded at the time of enrolment was associated with a 19% risk of high FRS (*vs*. low) (proportional OR=1.953, 95% CI 1.429-2.669; *p <*.0001). The EDSS score showed no association with moderate FRS (*vs*. low) (proportional OR=0.610, 95% CI .249-1.536, *p* =.294).

No association was found with disease activity in the year before or with other variables considered (Table **3**).

## 4 DISCUSSION

In our cross-sectional monocentric study, the disability reported as a patient-reported outcome (PRO) through PDSS was associated with a high FRS. Additionally, a higher EDSS score was associated with a high FRS.

Several large studies have revealed the high occurrence of cardiovascular disease in patients with MS, demonstrating that the risk of ischemic heart disease, stroke, or heart failure is significantly increased in this population, although with controversial results [8, 26].

Our results are consistent with previously published data on FRS in MS patients and disability accrual [10, 14, 27].

Additionally, our study was the first in the MS field to explore an association with a PRO, the PDSS. Overall, the PDDS is a promising PRO for disability that is simple and reliable [17, 18]. FRS has been used on MS patients’ population, but no studies have focused on quality of life or on the patients ‘disease perception. As known, PROs reflect the experiences that patients have in relation to their treatment or condition. People with MS are the foremost experts on what it means to live with this disease, the impact of treatment, and the critical aspects of their lives that a new drug or rehabilitation intervention must address. Comorbid conditions are also associated with decreased quality of life in MS [19]. The higher incidence of cardiovascular comorbidity compared with age-matched healthy controls may influence low-, mid-and long-term clinical outcomes, namely, greater general disability status, worse physical outcomes, higher depression scores, cognitive aging, and low satisfaction with quality of life [11].

The association between PDDS and EDSS has been investigated previously, providing real-world evidence that PDDS can accurately assess disability in MS [16].

Our findings were further supported by the correlation observed between FRS and the EDSS recorded at the time of enrolment. A study by Moccia *et al*. suggested that the FRS, evaluating global cardiovascular health by the interaction amongst different risk factors, relates to MS disability, severity, and course [10]. Specifically, linear regression analysis showed a direct relationship between FRS and EDSS (*p <* 0.001) [10].

A retrospective report on 251 MS patients showed that a one-point increase in the FRS was associated with a 19% higher risk of reaching EDSS 6.0 and a 62% higher risk of DMT escalation [27].

Another report by Marrie *et al*. focused on FRS and brain volumes. High FRS was associated with lower brain volumes in persons with MS at baseline and with brain volume loss over time also after further adjustment for age, gender, and use of disease-modifying therapy [14].

Our results underscore the importance of PDDS in daily clinical assessment and demonstrate how vascular comorbidities can impact not only objectively measured disability but also the perception of the disease. The study has several limitations.

Firstly, the observational nature and a monocentric (single-center) design in the study could introduce certain biases and confounding factors [28]. Observational studies rely on data collected in real-world settings, and this approach may be subject to biases such as selection bias (due to how participants are chosen) or information bias (due to data collection methods). Additionally, the monocentric design limits the generalizability of findings to broader populations, as the study is conducted at a single center with specific patient characteristics and practices. Determining how well the study sample represents the entire population of individuals with MS is challenging. The sample recruited for the study may not accurately reflect the diversity of patients with MS in terms of demographics, disease severity, or treatment patterns. This lack of representativeness could limit the external validity of study findings and may affect the applicability of results to a wider population.

Also, we only measured cardiovascular risk during daily clinical practice and no longitudinal data have been collected, and FRS is a long-term predictive score.

Additionally, information on other cardiovascular comorbidities or specific drugs prescribed was not available. In conclusion, the FRS demonstrated an association between patients ‘perception of the disease as indicated by the PDDS and disability as assessed by the EDSS score.

Given these considerations, it becomes essential to conduct further research using larger and more diverse cohorts. This approach can help validate initial findings, minimize biases associated with study design, and enhance the generalizability of results to a broader range of individuals with MS.

Careful attention should be given to addressing and treating cardiovascular risk factors, in addition to implementing lifestyle changes, when managing MS in the long term.

## AUTHORS’ CONTRIBUTIONS

It is hereby acknowledged that all authors have accepted responsibility for the manuscript's content and consented to its submission. They have meticulously reviewed all results and unanimously approved the final version of the manuscript.

## Figures and Tables

**Table 1:** Demographical and clinical characteristics of the investigated cohort.

**-**		**Summary**	**Whole Cohort**
		**Statistics**	**n=229**
**Sex**	Female	No. (%)	163(71.2)
**Age**	Years	Mean (SD)	40.4 (12.6)
**Disease duration**	Years	Median (IQR)	9 (3-6)
**EDSS (index date)**		Median (IQR)	2 (1.5-3.5)
**PDDS**		Median (IQR)	2 (2-3)
**Active patients in the year before index visit**		No. (%)	45(19.6)
**Patients naïve to DMTs**		No. (%)	154 (67.3)
**Patients switching from other DMTs**		No. (%)	75 (32.7)
**Smokers**		No. (%)	53 (23.1)
**Patients with hypertension**		No. (%)	76 (33.2)
**Patients with dyslipidaemia**		No. (%)	25 (10.9)
**Patients with diabetes**		No. (%)	45 (19.6)
**Overweight patients**		No. (%)	75 (32.7)
**Obese patients**		No. (%)	21 (9.1)
**FRS**		No. (%)	-
*Low risk*		-	97(42.3)
*Intermediate risk*		-	84 (36.7)
*High risk*		-	48 (21)

**Abbreviations:** SD: Standard Deviation, IQR: Interquartile range, Q1, and Q3 were reported, N, number; DMT, disease-modifying therapy; EDSS, Expanded disability status scale; FRS, Framingham risk score; PDSS, patients determined disease step scale.

**Table 2:** Framingham risk score: Univariable ordinal logistic model.

Variables**	**Low Risk ** **(n = 97)**	**Intermediate Risk ** **(n = 84)**	**High Risk ** **(n= 48)**	*p*-value*
**Age**	39.8 ± 12.8	41.4 ± 11.7	42.9 ± 12.9	.448
**Female, n (%)**	68 (71.3)	58 (69)	67(68.8)	.255
**Disease Duration (years)**	8 (2-8)	9 (3-7)	9 (3-6)	.377
**Naive n (%)**	31 (32.4)	27 (31.3)	17 (32.3)	.313
**Active patients in the year before index visit, n (%)**	20 (20.3)	16 (18.1)	9 (19.2)	.755
**EDSS-index date median [IQR]**	1.5 [1.0-2.0].	1.5 [1.0-2.5]	3.0 [2.0-3.5]	.000^αβ^
**PDDS score-index date median [IQR]**	2 [2-3]	2 [2-3]	3 [2-4]	.000 ^αβ^

**Note:** *via Anova and Chi-squared test; **data are reported as mean ± standard deviation when otherwise specified. ^α^Post-hoc high-risk versus low risk, ^β^Post-hoc high-risk versus intermediate risk.

**Abbreviations:** IQR: Interquartile range, Q1 and Q3 were reported. N, number; EDSS, Expanded disability status scale; FRS, Framingham risk score; PDSS, patients determined disease step scale.

**Table 3:** Framingham risk score: Univariable ordinal logistic model.

Independent Variable*	**Univariable Analysis**	
	**Prop OR (95% CI)**	***p*-value**
**Sex^1^**	.591 (.237-1.472)	.258
**Disease duration**	.550 (.450-1.552)	.144
**Naïve/switchers**	1.390 (.517-3.739)	.514
**EDSS (index visit)**	1.953 (1.429-2.669)	<.0001
**Active patients in the year before the index visit**	.953 (.444-2.044)	.901
**PDDS score (index visit)**	2.426 (1.660-3.545)	<.0001

**Note:** *For dichotomic variables the first category (0=no) was used as reference.

^1^Male sex was used as reference.

**Abbreviations:** N, number; EDSS, expanded disability status scale, PDDS patients determined disease steps, Prop: proportional; OR: Odds Ratio.

## Data Availability

Anonymised data will be shared upon request from any qualified investigator for the sole purpose of replicating procedures and results presented in the report, provided that data transfer is in agreement with EU legislation on the general data protection regulation.

## LIST OF ABBREVIATIONS

BMI: Body Mass Index
CNS: Central Nervous System
DMT: Disease-modifying Treatment
FRS: Framingham Risk Score
MS: Multiple Sclerosis
PDDS: Patient Determined Disease Steps
PRO: Patient-reported Outcome
